# Conservative Treatment of Splenic Haematoma After Colonoscopy: A Case Report

**DOI:** 10.7759/cureus.10531

**Published:** 2020-09-18

**Authors:** Beatrice D'Orazio, Bianca Cudia, Guido Martorana, Gaetano Di Vita, Girolamo Geraci

**Affiliations:** 1 Surgery, University of Palermo, Palermo, ITA; 2 Surgical, Oncological and Stomatological Sciences, University of Palermo, Palermo, ITA; 3 General and Oncological Surgery, Fondazione G.Giglio Cefalù, Cefalù, ITA

**Keywords:** endoscopy, colonoscopy, spleen, haematoma, traumatic injury

## Abstract

Colonoscopy is a routine procedure performed worldwide, nevertheless, a small risk of splenic injury, often under-estimated, is still present. As a matter of fact, the diagnosis may be delayed, leading to a rising risk of morbidity and mortality. This paper describes a case of conservative treatment of colonoscopy-associated splenic injury.

A 57-year-old woman presented with worsening pain in the upper left abdominal quadrant; she had radiation therapy to the ipsilateral subscapular region, and a diagnostic colonoscopy 18 hours earlier.

The computed tomography (CT) scan revealed splenic laceration without signs of hemoperitoneum. Because of the hemodynamic stability of the patient, successful conservative treatment and serial controls of the blood and hemodynamic parameters were adopted.

Even if rare splenic injury during colonoscopy is associated with significant morbidity and mortality. A high degree of clinical suspicion is essential to achieve a prompt diagnosis as well as an early surgical evaluation.

The nonoperative approach is usually taken in patients with no intraperitoneal bleeding, a closed subcapsular haematoma and a stable hemodynamic status.

## Introduction

Colonoscopy is commonly performed for diagnosis and treatment of benign and neoplastic pathology, as well as for colorectal cancer screening [1]. Although colonoscopy is generally safe and well tolerated, nearly one-third of patients complain of transient gastrointestinal symptoms, such as bloating (25%), abdominal discomfort (10.5%), diarrhea (6.3%), nausea (4%), and self-limiting bleeding (3.8%) [1]. Moreover, there is a small but significant risk of splenic injury that is often under-recognized. This kind of injury is often under-estimated, therefore the diagnosis is late, causing a rise in the morbidity and mortality rate [2].

Here we report a case of splenic haematoma after colonoscopy that was managed conservatively and successfully.

## Case presentation

A 57-year-old woman, already undergone several years ago to hysterectomy for multiple leiomyomas and laparoscopic cholecystectomy, came to our observation for worsening pain in the upper left abdominal quadrant, with radiation therapy to the ipsilateral subscapular region.

The patient underwent, about 18 hours before, to diagnostic colonoscopy in analgosedation for evaluation of chronic anaemia. The colonoscopy was uneventful and did not reveal a cause of her chronic anaemia.

Before colonoscopy, her haemoglobin was 10.8 g/dL and systolic blood pressure 120 and 85 mmHg with 76 beats/minute. The patient did not report enterorrhagia, hematemesis or melena.

In our examination the abdomen was soft, systolic blood pressure was 110/80 mmHg with 88 beats/minute; the haemoglobin was 9.5 g/dL and the ultrasound (US) examination was not diagnostic for the splenic injury because of the intestinal meteorism.

**Figure 1:**
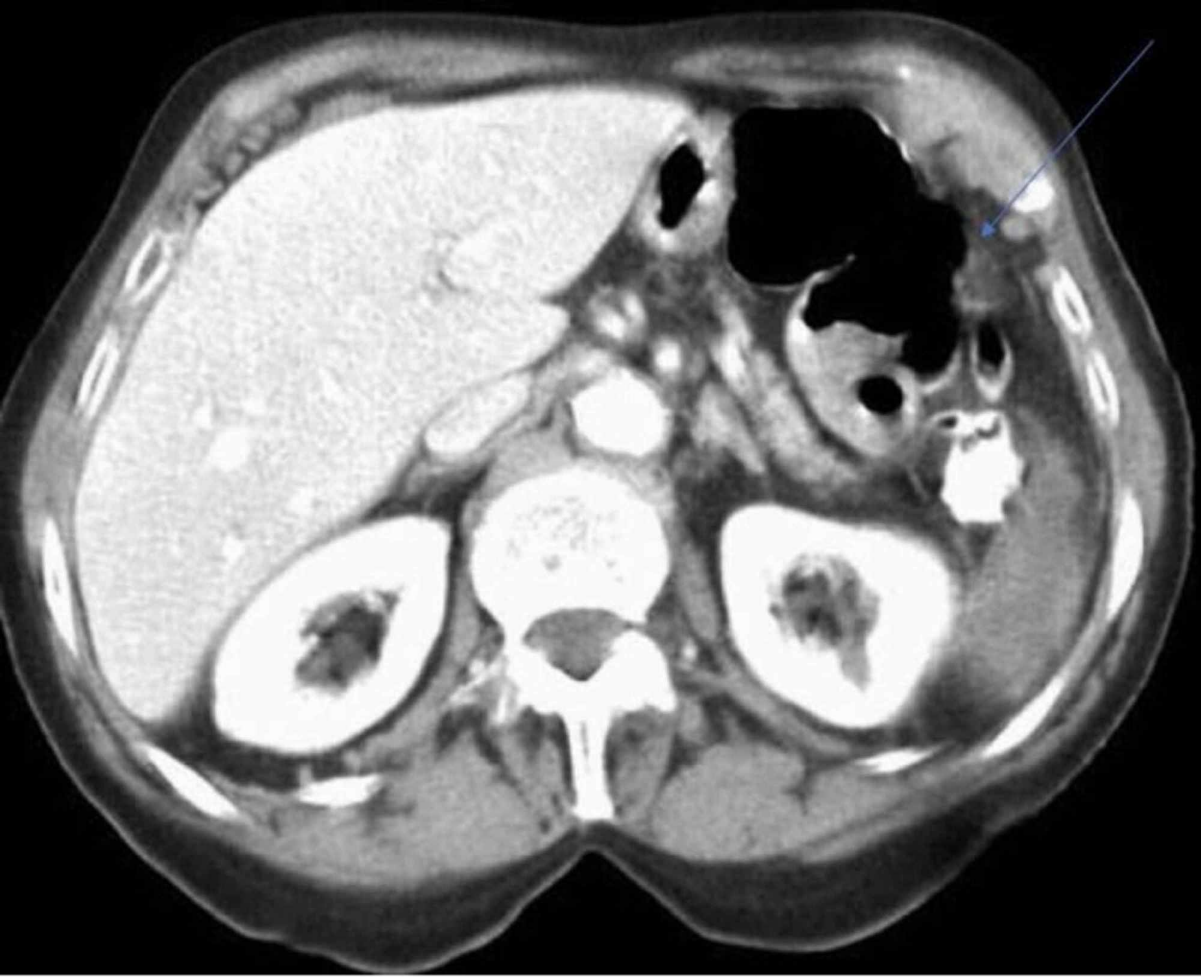
Abdominal CT scan frame showing the splenic injury caused during the colonoscopy: grade II splenic laceration without signs of hemoperitoneum; anterior capsule of the spleen was densely adherent to the splenic flexure of the colon and partially avulsed.

The computed tomography (CT) scan revealed grade II splenic laceration without signs of hemoperitoneum where the anterior capsule of the spleen was noted to be densely adherent to the splenic flexure of the colon and partially avulsed (Figure 1).

The patient was promptly hospitalized and conservative treatment was undertaken. The hemodynamic parameters remained stable, the pain was reduced in intensity and the US control did not show the hematoma increase in dimension. The patient was discharged after three days and the eight-month follow-up showed the complete resorption of the haematoma without remnants.

## Discussion

In a systematic review of 12 studies including 57,742 screening colonoscopies, the rate of serious adverse events was found to be as low as 0.28% [3]. Another study based on a database of 2.3 million colonoscopies found that a complication requiring hospitalization occurred in 1.9 per 1000 colonoscopies [4].

Splenic injury after colonoscopy procedure is rare but a serious complication. From 1974 to 2012, a total of 103 cases have been reported [5]. Jehangir et al. reported 172 cases described in the literature before 2015 [6]. Among those, there were more females (64-76.5%), median age was 65 years (range, 29-90 years), and most of the colonoscopies were performed without difficulty (66.6%) [5].

The overall incidence of splenic injuries ranges from 1 in 100,000 to 1 in 6,387 colonoscopies [7].

In the US, we estimate that in 2004 we performed 22.4 million colonoscopies [8]. So taken into consideration the incidence of splenic injury of 1 in 100,000 exams, we can imagine that 224 splenic injuries have been caused only in 2004. Consequently, in accordance with various authors, we think that splenic injuries during colonoscopies are under-detected and under-estimated; the possible reason for this includes the misattribution of pain to gas discomfort, particularly in haemodynamically stable patients [4-6,9,10].

The mechanism for splenic trauma during colonoscopy is yet to be fully understood - postulation is the excessive traction on the splenocolic ligament and/or on splenocolic adhesions, secondary to previous abdominal surgery or intraabdominal inflammatory processes, or a direct blunt trauma when navigating the colonoscope through the splenic flexure [4,6].

Known risk factors for splenic trauma also include older age, less experienced endoscopists, being female, polypectomy and biopsies during the procedure [4]; other contributing factor to splenic injury includes the blind advancement of the endoscope past the splenic flexure to straighten the left colon.

Preventive measures are good colonoscopy technique to avoid loop formation and left lateral position of the patient to reduce the risk of splenic injury after colonoscopy [11]. The use of anticoagulant medications has been reported in some cases, but does not actually correlate with splenic injury [12,13].

Nearly 67% of patients presented within 24 hours of colonoscopy with complaints ranging from abdominal pain to dizziness. The most common symptom is left upper quadrant pain (95%), and CT scan was found to be the most sensitive and specific tool for diagnosis (98.5%) and grading the extent of splenic injury. Abdominal pain either generalized or in left upper quadrant is the chief complaint in most patients (85%), only 15% presented with signs of hemodynamic instability such as dizziness and syncope [2].

According to the American Association for the Surgery of Trauma (AAST), splenic injuries are classified as grade I - subcapsular hematoma <10% of surface area, capsular laceration <1 cm depth; grade II - subcapsular hematoma 10-50% of surface area, intraparenchymal hematoma <5 cm in diameter, laceration 1-3 cm depth not involving trabecular vessels; grade III - subcapsular hematoma >50% of surface area or expanding, intraparenchymal hematoma >5 cm or expanding, laceration >3 cm depth or involving trabecular vessels, ruptured subcapsular or parenchymal hematoma; grade IV - laceration involving segmental or hilar vessels with major devascularization (>25% of spleen); grade V - shattered spleen, hilar vascular injury with devascularized spleen [14].

In Piccolo et al. review of the literature only 24% of cases were managed conservatively while 70% was treated by splenectomy as a definitive management [5]. Severe complications could be associated with this latter procedure, five deaths in patients with splenic injuries [9].

## Conclusions

Colonoscopy is a safe procedure hardly ever burdened by splenic injuries, therefore their management is independent from the colonoscopy indication. Nevertheless, the treatment of this complication should be tailored on the type of injury, reserving the surgical approach to unstable patients with spleen rupture, which leads to a significantly higher mortality rate.
